# Immunological Aspects of Human Reproduction

**DOI:** 10.1155/2012/408329

**Published:** 2011-12-07

**Authors:** Raivo Uibo, Andres Salumets, Gilbert Faure

**Affiliations:** ^1^Immunology Group, IGMP and Centre of Excellence for Translational Medicine, University of Tartu, 50411 Tartu, Estonia; ^2^Competence Centre of Reproductive Medicine and Biology, Tiigi 61b, 50410 Tartu, Estonia; ^3^Department of Obstetrics and Gynecology and IGMP, University of Tartu, 51014 Tartu, Estonia; ^4^Laboratory of Immunology, Faculty of Medicine, Université Henri Poincaré and Nancytomique, CHU Nancy-Brabois and EA 4369 RHEM, 54500, Vandoeuvre lès Nancy, France

The scientific community of immunologists has been interested in immunological aspects of reproduction since the beginning of last century. Although many questions of reproductive immunology still remain unanswered, several important results have been obtained during recent years. Most importantly, the topic has achieved more wider impact than before. Presently, immunology of human reproduction involves not only the interaction between the maternal immune system and the fetus-placenta status, but also many diverse aspects of reproduction ranging from human gametogenesis to immuno(epi)genetic aspects of diseases of the reproductive system of females and males. Since fertility rates are falling in many countries implying wider usage of various assisted reproduction technologies to overcome reproductive failure, the topic of immunology of human reproduction is receiving increasing attention. Conversely, immunology journals are publishing more papers on every aspect of the immunology of reproduction. The completion of this special issue of Clinical and Developmental Immunology is a good example. 

The present issue of this journal is presenting six reviews and eight original papers on diverse immunological aspects of human reproduction. 

The first review of S. J. Chen et al. concentrates on abnormalities in the maternal-fetal immunological relationship and the current immunological therapeutic strategies for pathological disorders developing during pregnancy. In the next review paper, K. H. Kikkatalo et al. point out the importance of autoimmune reactions in the development of female infertility and unravel the role of immune reactions against follicle stimulating hormone (FSH) in modulation of female reproductive function. In another review related to hormones and immune reactivity interactions, N. Vrachnis et al. present data on the role of progesterone and corticotropin-releasing hormone (CRH) in myometrium and show their interaction with the immune system during labor. Review paper of M. D. Benson discusses the immunology of amniotic fluid embolism that is one of the leading causes of maternal mortality and morbidity in many countries. In their review about placental IgG transfer in healthy and pathological pregnancies, P. Palmeira et al. analyse the factors participating in IgG transfer. D. V. Vujaklija et al. have chosen to analyse the mechanisms related to cell death at the maternal-fetal interface. In their paper, cytotoxic cells as well as the role of granulysin are under deep scrutiny.

In the original research paper series, the study of J. Calleja-Agius et al. presents the results of the influence of abnormal placental karyotype on inflammatory response evaluated by tumor necrosis factor (TNF) alpha, TNF receptors, and interleukin-10 measurements within villous tissue and blood from women with miscarriage. In the next paper by S. Cardaropoli et al., the fetal growth is studied in connection with macrophage migration inhibitory factor (MIF) and its role in preeclampsia pathogenesis is presented. A. Sarapik et al. bring new data on levels of cytokines, chemokines, and other inflammatory markers in the follicular fluid of patients with different in vitro fertilization (IVF) outcome. The paper of R. Raghupathy et al. presents data about production of pro- and anti-inflammatory cytokines by peripheral blood mononuclear cells stimulated with trophoblast antigens. These authors show that in case of intrauterine growth restriction, a proinflammatory bias exists in comparison with normal pregnancy. In the paper of M. T. Ahlen et al., the impact of the maternal anti-human platelet antigen 1a (HPA1a or GPIIIa) antibodies in determination of neonatal alloimmune thrombocytopenia is analyzed according to maternal ABO genotypes. W. X. Xu and coauthors are presenting their results on the characterization of B-cell epitopes on human zona pellucida glycoprotein-3, the sperm receptor protein known to have a critical role in fertilization. C. Agostinis et al. have focused their work in assessing the role of mannose binding lectin (MBL) in preeclampsia where this immunologically active substance could also contribute to the endovascular invasion of trophoblast cells. In their paper, W. Zaigui et al. show that functional polymorphism of the gene of Foxp3, a transcription factor involved in regulatory T-cells function, may confer susceptibility to unexplained recurrent spontaneous abortion.

*Raivo Uibo* *Andres Salumets* *Gilbert Faure*

